# A numerical study of the aerosol behavior in intra-acinar region of a human lung

**DOI:** 10.1063/5.0024200

**Published:** 2020-10-01

**Authors:** Dogan Ciloglu

**Affiliations:** Vocational School of Higher Education, Atatürk University, Erzurum 25240, Turkey

## Abstract

The determination of the particle dynamics in the human acinar airways having millions of alveoli is critical in preventing potential health problems and delivering therapeutic particles effectively to target locations. Despite its complex geometrical structure and complicate wall movements, the advanced calculation simulations can provide valuable results to accurately predict the aerosol deposition in this region. The objective of this study was to numerically investigate the aerosol particle transport and deposition in the intra-acinar region of a human lung for different breathing scenarios (i.e., light, normal, and heavy activities) during multiple breaths. Idealized intra-acinar models utilized in this study consisted of a respiratory bronchial model, an alveolar duct model, and an alveolar sac model. The particles with 5 *μ*m in diameter released from the inlet of the model were tracked until they deposited or escaped from the computational domain. The results showed that due to the rhythmic alveolar wall movement, the flow field was divided into two regions: one is the low-speed alveolar flow and the other is the channel flow. It was found that the chaotic acinar flow irreversibility played a significant role in the aerosol transport in higher generations. During the succeeding breaths, more particles deposited or escaped to the relating acinar generation and reached the more distal regions of the lung. The number of particles remaining in the suspension at the end of the third cycle ranged from 0.016% to 3%. When the mouth flow rate increased, the number of particles remaining in the suspension reduced, resulting in higher deposition efficiency. The total deposition efficiencies for each flow rate were 24%, 47%, and 77%, respectively. The particle simulation results also showed that more breathing cycle was required for full aerosol particle deposition or escape from the model. In addition to the alveolar wall motion, the type of breathing condition and breathing cycle had a significant effect on the accurate prediction of the aerosol deposition in the intra-acinar region of the human lung.

## I. INTRODUCTION

The inhaled air may carry many toxic particles or pharmaceutical aerosols to the human respiratory system in addition to oxygen. During inhalation, the fresh air inhaled through the mouth or the nose is transported to the distal zones of the lungs (i.e., the acinar region), while the waste air leaves the body by following the same path during exhalation. The acinar region is surrounded by a large number of small blood vessels called capillaries, where the gas exchange occurs.^1–4^ Thus, determining the particle dynamics in this region is a critical issue to prevent potential health problems or to deliver effectively therapeutic particles to target locations.

Many studies in the literature showed that some biological (i.e., lung morphology and breathing conditions) and physical factors (i.e., aerosol properties and deposition mechanisms) have a vital role in determining the airflow and the fate of inhaled aerosols in the human respiratory tract. For example, Salma *et al.*^5^ investigated the effect of physical exertion on aerosol deposition, deposition rates, and surface doses in the whole respiratory system. They reported that the level of physical exertion during a single breath plays a significant role in the fraction of particle deposition. Hofmann *et al.*^6^ numerically investigated the gravitational deposition patterns of 10 *μ*m particles in the asymmetric bifurcation airway model representing the generations 15 and 16 for low and heavy physical activity breathing conditions. They reported that the direction of gravity is significant for the deposition efficiency (DE). Kolanjiyil and Kleinstreuer^7^ employed a new whole-lung-airway model (WLAM) to simulate the airflow and particle behavior for different inhalation/exhalation scenarios (normal, light, and heavy activities that correspond to the inhalation flow rates of 15 l/min, 30 l/min, and 60 l/min, respectively, and different particle diameters in the size range 0.93 *µ*m–30 *µ*m). The authors suggested the use of their whole-lung model for analyzing the human exposure to toxic particulate matter or estimating pharmacological effects of administered drug-aerosols.

Harrington *et al.*^8^ developed the first 3D bifurcation model of a fully alveolated duct representing the generations 18–22 with rectangular alveoli compartments. They indicated that the orientation of the models of acinar airways strongly influenced the aerosol deposition of 1 *μ*m–5 *μ*m diameter particles. A more complex 2D multi-generation structure of alveolated ducts was presented by Darquenne.^9,10^ He investigated both the flow field and the trajectories of 2 *μ*m-diameter particles in the alveolar region of the human lung and reported the heterogenic deposition based on the gravity vector. In order to estimate the regional particulate deposition during multiple breaths, a simple semi-empirical model for whole lung aerosol bolus dispersion was presented by Park and Wexler.^11^ They stated that the alveolar wall motion is important to determining the enhancement of particle deposition in the more proximal pulmonary regions. Ertbruggen *et al.*^12^ compared the numerical predictions and experimental results of the acinar flow in a 3D alveolated bend geometry with rigid walls. They indicated that the numerical results were agreed well with those experimentally obtained. Darquenne *et al.*^13^ used the first 3D model of a fully alveolated duct with moving walls and simulated the transport and deposition of the particles ranging from 1 *µ*m to 5 *μ*m in diameter in the acinar region of the lung. They performed the simulations on the horizontal duct and with the gravity vector acting downward. It was shown that more particles deposited for the moving wall model as a result of the increase in the convective transport in alveolar cavities, especially for small particles. On the other hand, the authors did not address the effect of gravity orientation on deposition.

Kumar *et al.*^14^ simulated the flow analysis in the acinar region using a 3D honeycomb-like polygonal model. Their numerical results showed that the flow into and out of the alveoli was formed by the alveolar wall motion, and it was characterized by the flow entrainment regions. Ma and Darquenne^15^ simulated the airflow and depositions of 1 *µ*m and 3 *μ*m aerosol particles in models of the human alveolar sac and terminal acinar bifurcation under rhythmic wall motion for two breathing conditions. Monjezi *et al.*^16^ numerically predicted the flow and particle deposition for 39 different particle sizes in a fully coupled 1D–3D whole lung model. Żywczyk and Moskal^17^ investigated the fluid flow and the aerosol dynamics in a rhythmically expanding and contracting 2D human alveolus model. The results indicated that the mechanical properties of the tissue and the gravity play a significant role in the fluid flow and the deposition of aerosol particles. They also depicted that the smaller particles deposited for a longer period of time because these particles remain in the fluid for longer compared to bigger ones. Khajeh-Hosseini-Dalasm and Longest^18^ reported the flow field dynamics and particle transport on a moving wall model of a pulmonary acinus at six different gravity angles. Contrary to the previous study,^17^ surprisingly, the authors depicted that the total acinar deposition was not affected by the gravity orientation angle. Georgakakou *et al.*^19^ conducted an analytical study on a simplified model to predict the alveolar deposition for particle diameters d_p_ ≥ 0.1 *μ*m. More recently, Talaat and Xi^20^ examined the effects of various physiological factors including the wall motion modes, particle size, alveolus orientation, breathing frequency, and depth on the flow and particle deposition in the expanding–contracting terminal alveoli. They proposed a correlation for particle deposition in the terminal alveoli that captured the separate contributions on the gravitational sedimentation and periodic wall motion. In order to accurately predict the total, segmental, and regional particle transport through the lung airways, a realistic whole-lung airway model having the alveolar movement was developed by Kolanjiyil and Kleinstreuer.^21^ They stated that the particle behavior depends on the lung-airway geometry, particle characteristics, and inhalation flow frequency.

The particle deposition models in the literature mentioned above may be grouped into two main headings: the whole and local lung models. While the flow and particle deposition equations for the whole lung models^22,23^ are solved by analytical equations, they are solved numerically by computational fluid dynamics (CFD) methods in the case of local scale models, i.e., the pulmonary region.^24^ In these models, the static alveoli geometries^9,25–29^ or the simplified acinus models^3,30^ have been generally employed by the researchers. However, only a few numerical studies have focused on both developing realized alveolar airway model and considering some biological or physiological factors, such as the breathing condition, breathing cycle, and alveolar wall motion.^6,10,31^ Detailed knowledge of inhaled toxic particles or pharmaceutical aerosol transport and deposition in this region is crucial in evaluating the effects of toxicants or the results of the treatment. Thus, more studies may help in a better understanding of this phenomenon. In this study, idealized intra-acinar models with rhythmic alveolar wall motion representing the generations 17, 18, and 23 have been established to simulate the airflow and aerosol transport. The mouth inlet flow rates of 15 l/min, 30 l/min, and 60 l/min for people under light, normal, and heavy activities, respectively, are applied to perform unsteady inspiration and expiration during multiple breathing cycles.

## II. METHODS

### A. The geometrical models

**FIG. 1.**
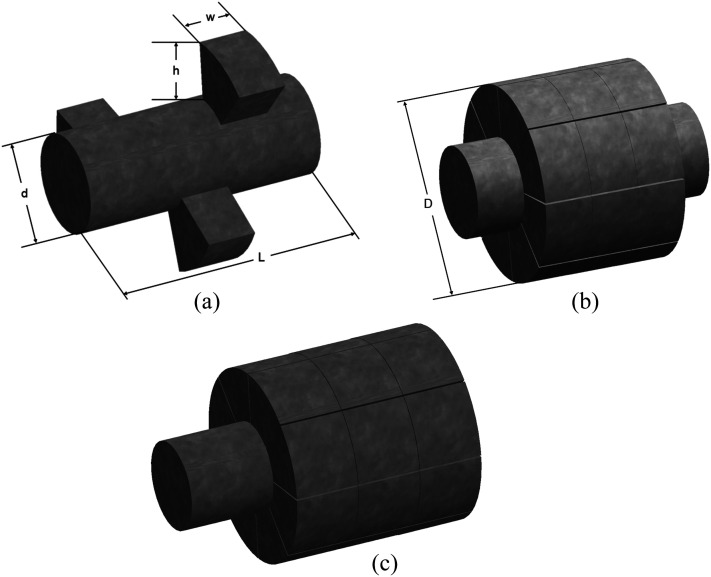
3D models representing the intra-acinar region of the human lung: (a) the respiratory bronchiole, (b) the alveolar duct, and (c) the alveolar sac.

The respiratory tract is divided into three regions: the upper part, the distal part, and the acinar part. The acinar region consists of the respiratory bronchioles, the alveolar ducts, and the alveolar sacs. An acinar unit contains about 15 000 alveoli and not all can be simulated in a logical CFD model.^32^ In this study, an alveolated intra-acinar model has been developed to accurately predict the total acinar deposition, as seen in Fig. 1. The geometric characteristics of the respiratory bronchiole (G17), the alveolar duct (G18), and the closed-end alveolar sac (G23) are similar to those proposed by Yeh and Schum,^33^ Harrington *et al.*,^8^ and Ma and Darquenne,^15^ respectively. Alveoli have been assumed to have a rectangular shape, as used in the study Harrington and co-workers.^8^ Table I illustrates the geometric parameters of the models used in this study. There was a minimum distance between the alveoli surrounding the model surfaces to enable the wall movement. The alveolar wall motion during expansion/contraction creates the airflow within the alveolus and derives particle transport in the deeper parts of the lung, containing the acinar region.^15^

**TABLE I.** Geometric parameters of the models.

	Generation number		
Parameter (cm)	G17	G18	G23
D	0.0500	0.0470	0.0206
D	0.2200	0.1000	0.0575
H	0.0300	0.0265	0.0169
L	0.1200	0.0920	0.0832
W	0.0250	0.0250	0.0150

### B. Airflow equations

Table II shows the respiratory parameters for an average human adult under different breathing conditions. Since the Reynolds numbers at the inlet of airway generations range from 0.01 to 1.16 (see Table II), the airflow in this region is laminar. Therefore, the airflow is described by solving the following 3D governing transport equations for the incompressible flow on a commercial finite-volume based program, i.e., Fluent 19.0 (ANSYS, Inc., Canonsburg, PA),

$$\nabla \cdot \vec{u}=0,$$ (1)

$$\rho \frac{d\vec{u}}{dt}+\rho \left(\vec{u}\cdot \nabla \right)\vec{u}=-\nabla p+\mu \nabla^{2}\vec{u}+\rho \vec{g},$$ (2)

where $\vec{u}$ denotes the velocity vector ($\vec{u}=\left[u,v,w\right]$), *p* is the air pressure, *ρ* is the density, *t* is the time, and *μ* is the dynamic viscosity. In the simulations, the physical properties of the air are at 37 °C.

**TABLE II.** The respiratory physiological parameters used in the models for an average human adult.

Airway generation	Breathing conditions	Flow rate (l/min)	Re
G17	Light	15	0.29
	Normal	30	0.58
	Heavy	60	1.16
G18	Light	15	0.15
	Normal	30	0.31
	Heavy	60	0.62
G23	Light	15	0.01
	Normal	30	0.02
	Heavy	60	0.04

### C. Particle transport equations

In this study, the interactions between particles are neglected since the particle flow is typically dilute. For the particles less than 5 *μ*m in diameter, the effect of Brownian diffusion is also negligible.^34^ Moreover, the possible particle size changes are not taken into account in the simulations. The no-slip boundary condition is applied at the wall boundaries. In order to predict the particle trajectories in the acinar region, the effects of the drag force and the gravitational force are considered, therefore,

$$m_{p}\frac{d{\vec{u}}_{p}}{dt}={\vec{F}}_{D}+m_{p}\left(\rho_{p}-\rho \right)\frac{\vec{g}}{\rho_{p}},$$ (3)

where ${\vec{F}}_{D}$ is the drag force for the spherical particles, which is defined as

$${\vec{F}}_{D}=\frac{1}{2}C_{D}A_{p}\left(\vec{u}-{\vec{u}}_{p}\right)\left|\vec{u}-{\vec{u}}_{p}\right|,$$ (4)

where *C*_*D*_ is the drag force coefficient given by

$$C_{D}=a_{1}+\frac{a_{2}}{Re_{p}}+\frac{a_{3}}{Re_{p}^{2}},$$ (5)

where *a*_1_, *a*_2_, and *a*_3_ are constants proposed by Morsi and Alexander.^35^ The particle Reynolds number is

$$Re_{p}=\rho \left|\vec{u}-{\vec{u}}_{p}\right|d_{p}/\mu \text{.}$$ (6)

The regional deposition efficiency (DE) of the aerosol particles in the human airways is calculated as

$$\text{DE}=\frac{number\;of\;deposited\;particles\;in\;a\;specific\;region}{number\;of\;particles\;entering\;the\;generation\;inlet}.$$ (7)

## III. NUMERICAL SETUP

In this study, the particle transport and deposition in the intra-acinar region of the human lung were numerically examined, assuming gravity in the negative *y*-direction. Three different flow rates, i.e., light (15 l/min), normal (30 l/min), and heavy (60 l/min) breathing conditions with tidal volume 0.5 l, were chosen for the simulations. One-way coupling was applied in the simulations, i.e., the particulate phase was assumed to have a negligible influence on the surrounding gas phase. The airflow in G17, G18, and G23 is solved for these inspiration flow rates, assuming a parabolic air velocity profile at the model entrance. All the calculations were conducted as double-precision to more accurately determine the airflow and the particle behavior. Three successive breathing cycles were conducted in the present simulations. The period of one breathing cycle was estimated to be 4 s (2 s inspiration and 2 s expiration, without pause), and therefore, the time for completing three breathing cycles was 12 s. It was assumed that both expansion and contraction followed the square wave inhalation profile. The alveolar wall motion was assumed to be isotropic spatially during respiration in all directions with the volume excursion of 25%. The inlet flow rates for G18 and G23 were calculated taking into account the flow lost in previous generations as a result of alveolar deformation.^14,21^

The aerosol particles used in the simulations have a spherical form and a density of 1100 kg/m^3^. The particle size (5 *μ*m) used in this study is the size of typical pharmaceutical drugs used in lung diseases. The larger of them do not reach the acinar region because of high inertial impaction.^20^ After the first breathing cycle (t = 0 s–4 s) set for just airflow simulation, 50 000 particles/s were released into the flow field with the continuous injection style at the beginning of the second breathing cycle (t = 4 s), and then monitored until they deposited or exited the model during multiple breaths. If a particle contacted with the model boundary, it was considered to be deposited and no longer considered in the calculations. During the third breathing cycle (t = 8 s–12 s), no new particles were put into to the computational domain, and the calculations were continued with the particles remaining in the suspension at the end of the expiration phase. The particles were assumed to have the same as that of air velocity at the inlet and have uniform distribution.

In order to minimize numerical uncertainties, some computations were performed and tested for time step and mesh independency. For example, a time step of 0.005 s was used, and the total deposition was 0.3% than that of a time step of 0.05 s. Thus, the time step in all simulations was fixed at 0.05 s. The transient simulation for each time step was assumed to converge when the residuals decreased below 10^−6^. On the other hand, the independence of the result from the mesh topology was provided by refining the mesh. For the alveolar sac model, a grid sensitivity study using three mesh configurations made of 349 913 nodes and 1 928 570 elements, 547 985 nodes and 3 054 643 elements, and 858 178 nodes and 4 838 219 elements was performed. Although the number of elements increases by a factor of ∼2.6, the difference in the total and regional deposition results was no more than 2%. Therefore, 995 248, 2 114 624, and 1 928 570 elements were adopted in all simulations for the respiratory bronchial, the alveolar duct, and the alveolar sac model, respectively. The computations were performed on a DELL workstation with 32 GB RAM and two 2.30 GHz Intel Xeon CPUs. The run-time of a transient simulation for three breaths lasted from 15 h to 32 h.

## IV. RESULTS AND DISCUSSION

### A. Validation

**FIG. 2.**
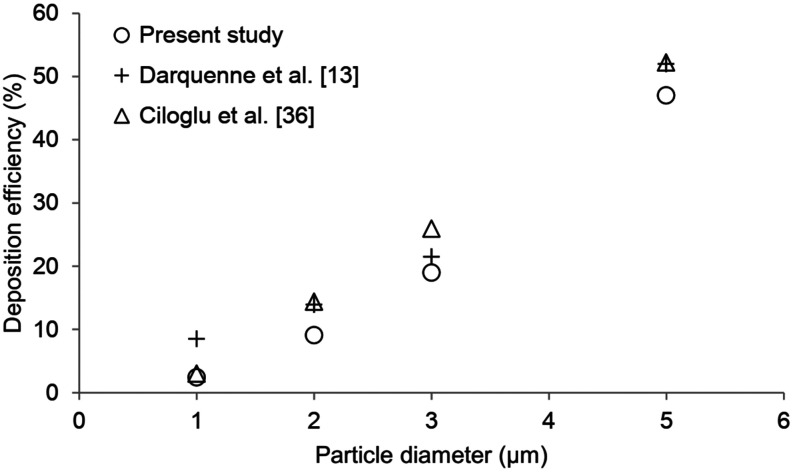
Comparison of the aerosol deposition fractions.

In this study, the particle transport and deposition in different breathing scenarios such as light (15 l/min), normal (30 l/min), and heavy (60 l/min) breathing conditions were numerically investigated. The computer simulation results have been validated with the numerical results of Darquenne *et al.*^13^ and Ciloglu *et al.*^36^ for an alveolar duct model (G18), the flow rate of 30 l/min (not including expiration), and particles with diameter between 1 *µ*m and 5 *μ*m. Figure 2 shows the comparison of the particle deposition fractions as a function of particle diameter in the acinar region. As seen in Fig. 2, the results of the present study have a good agreement between the numerical data. It can be considered that the present models are satisfactory to the 3D airflow analysis and aerosol dynamics in the intra-acinar region of the human lung.

**FIG. 3.**
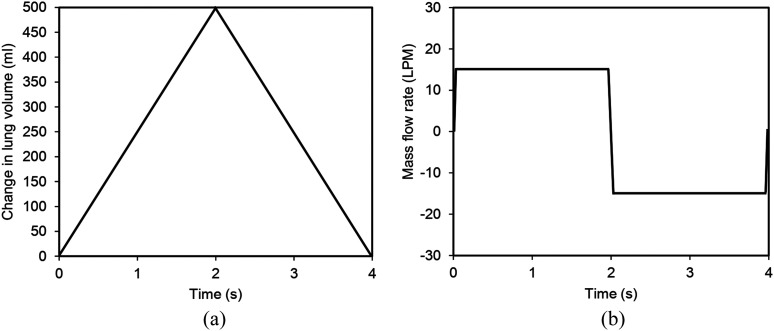
The simple breathing profile depictured in terms of (a) the change in the lung volume of 500 ml and (b) the corresponding mass flow rate of 15 l/min.

The models presenting the intra-acinar region of the human lung were used to simulate the airflow and the aerosol particle dynamics in different breathing scenarios with an inspiration/expiration period of 4 s (a tidal volume of 0.5 l). Figure 3 illustrates a simple breathing waveform profile for the light activity (i.e., a mouth flow rate of 15 l/min). For an average human adult with FRC of 3 l and total lung capacity 6 l, the alveolar enlargement is estimated to be ∼0.25.^30,34,37^

### B. Airflow results

**FIG. 4.**
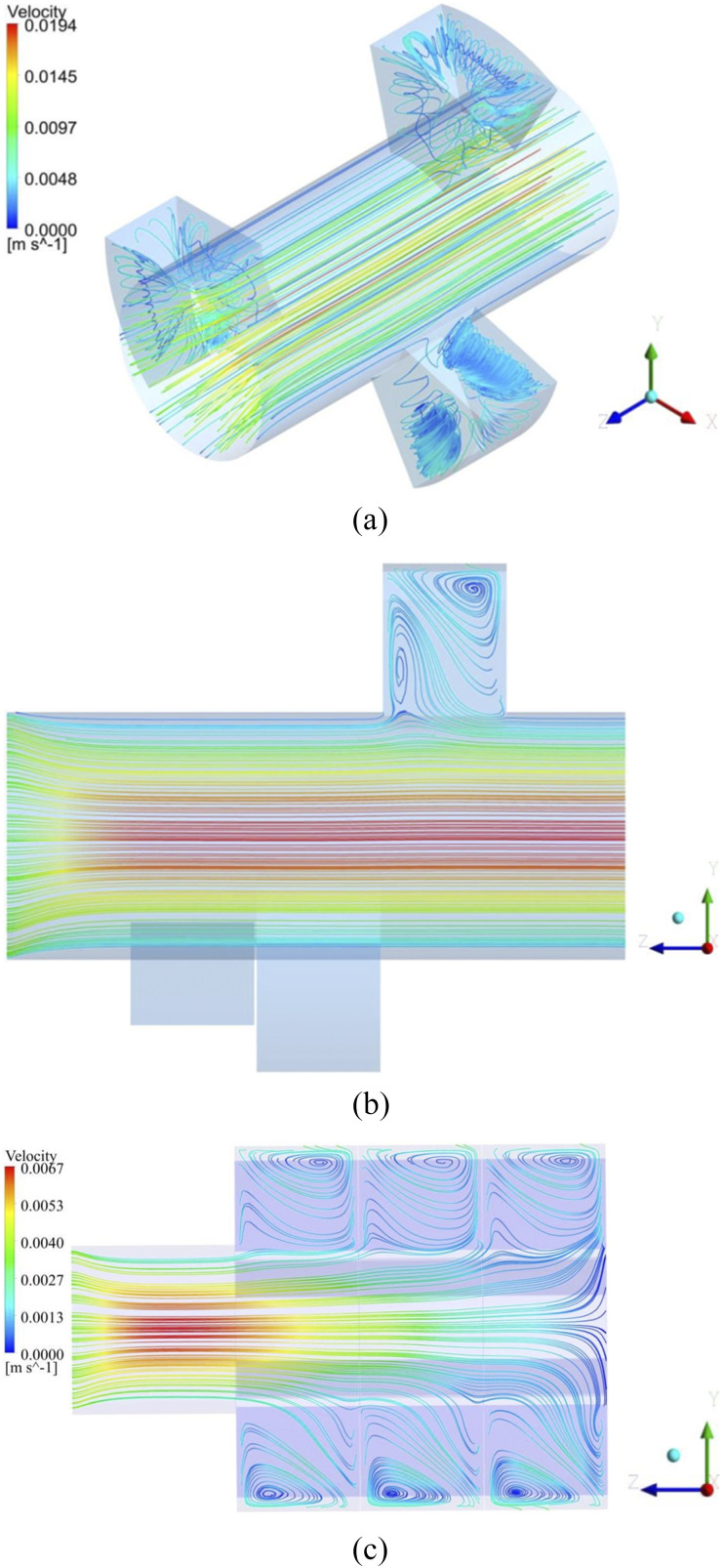
(a) 3D airflow pattern and 2D streamline pattern (b) in G17 for the flow rate of 15 l/min (Re = 0.29) and (c) in G23 for the flow rate of 60 l/min (Re = 0.04).

The intra-acinar 3D and 2D airflow patterns during inhalation for the flow rates of 15 l/min and 60 l/min, i.e., light and heavy breathing conditions, are shown in Fig. 4. As a result of the rhythmic expansion/contraction rate of alveoli and the pressure gradients in the outer flow, the separated flow zones were observed at the entrance to the alveoli. While the bulk flow continued along the lumen channel, some part of the fluid was directed into the alveoli (the alveolar flow), resulting in the recirculation flow regions [Fig. 4(b)]. The recirculation (or vortices) at the mouth of the alveoli was caused by the abrupt turn of the flow as it entered the alveolar cavity. This recirculating flow considerably manipulates the flow pattern.^38^ For the generations 18 and 23, when the Reynolds numbers increased, it was observed that the vortices formed at the mouth of the alveoli gradually shifted to the distal part of the alveolar cavity [Fig. 4(c)]. Therefore, the flow creates large recirculation regions in the downstream of the alveolar cavities. The separation region, on the other hand, highly affects the particle residence times in the alveoli.^38^ During the exhalation phase, the flow characteristics are almost the same as in the inhalation, except that the flow direction is reversed.

Zhang and Kleinstreuer^39^ analyzed numerically the unsteady airflow structures and micrometer-particle transport in a four-generation (G3–G6) airway model under normal breathing and high-frequency ventilation. They also depicted that the recirculation regions were formed in the downstream zone resulting in lower aerosol deposition compared to that of the lower flow rate case. They also declared that the flow asymmetry had little effects on the behaviors of aerosol particles during a moderate respiratory cycle. This is because, first, fewer particles go into the airway at low flow rates, and second, the time is required for particle deposition. The behavior of the aerosol particles is strongly dependent on the density of the secondary flow. At a low flow rate (or small secondary velocity), the particles followed the vertical flow more. At a high flow rate (or high secondary velocity), the particles moved away from the eddy centers to form the regions without particles (as shown in Fig. 7). During expiration, the particle motion is more complicated. In this case, however, the secondary flows are an important mechanism in determining the aerosol particle deposition.

Regarding the spatial growth of instability, the numerical studies have indicated that significant flow variations take place in the alveoli. Indeed, the flow phenomena in the circular channels that abruptly contract or expand have been the subject of many studies.^38–44^ It has been reported that at low Reynolds numbers, the flow instabilities have appeared near the expansion/contraction zone, especially. These flow instabilities are similar to the flow instabilities occurring in a stenosis. Mallinger and Drikakis^40^ numerically studied the unstable, three-dimensional, and pulsatile flow by stenosis and investigated the effects of instability on the velocity, wall shear stress, and vorticity fields. They reported that the instability in the separated flow region downstream of the stenosis caused significant asymmetries. Cantwell *et al.*^41^ numerically examined the transient linear dynamics in an axisymmetric expanding-pipe flow. For Reynolds numbers that do not cause any linear instability, they showed that the perturbations grew very strongly in the region of the separated shear layer lying downstream of the expansion. Choi *et al.*^42^ investigated the intra- and inter-subject variabilities of the inspiratory flow for two different flow rates in the airway models of two human subjects inspired by CT images. The researchers noted that the inspiratory flow characteristics depend on the shape, size, and flow conditions of the airway geometry. Varghese *et al.*^43^ investigated the steady and low-Reynolds-number pulsatile flow in stenosed tubes using a spectral-element method at different upstream and downstream sections of the tube. The authors stated that the stenotic flows may have flow separation, recirculation, and strong shear layers that can result in the periodic flow transitions in the stenotic region. Therefore, the presence of a stenosis can decrease the flow velocity through flow blockage. In the present case, as mentioned before, the vortices emerged from the near-entrance of the alveoli and gradually cover a large part of the alveoli [Fig. 4(b)].

The results also indicate that the velocity in the alveolar cavities is lower when compared to the channel flow, and the recirculating flows with varying densities in the alveoli are observed, as can be seen in Fig. 4(b). Berger and Jou^38^ investigated the flow characteristics in stenotic blood vessels. They expressed the presence of sites with low shear stresses or rapid changes in time and space occurring under some conditions such as unsteady flow, curved or bifurcated channels, junctions, branches, and the existence of sudden changes in the flow geometry. In the stenotic regions, the wall shear stresses can be greatly increased or decreased, and the upstream and downstream flow can be quite disturbed, thus resulting in flow separations or the recirculating flows, as seen in the present study. As the Reynolds numbers increase, on the other hand, the flow changes rapidly and the particles are washed out faster, resulting in shorter particle residence time. However, the researchers stated that the particle residence time was not related to the degree of stenosis of the channel.^38^ In order to investigate the effects of pulsatile flow on the post-stenotic flow, the fluid motion has been tracked for three mean Reynolds numbers just downstream of the idealized stenosis geometry by Jeronimo and Rival.^44^ The movement of the particles is strongly dependent on the pulsatile flow conditions. It has been shown that higher fluid velocities lower the particle residence time as the Reynolds number increases.

In general, the numerical simulations indicated that the low-Reynolds-number acinar flow has exhibited some features such as laminar-to-transitional flow transition, vortices, skewed axial velocity profiles, and secondary flow motions.^45–47^ The breathing patterns (or Reynolds numbers) determine the transitions between flow types. In the present study, it was also found that the weak flow irreversibility and vortices occurred within some alveoli, as mentioned before. This is due to the instability of the flow field in the pulmonary alveoli and is different from the recirculation caused by the viscous effects of the channel flow during respiration.^48^ These instabilities were attributed to the static properties of the alveolar fluid lining, non-uniform deformation of the lung tissue, and avalanche shocks that arose from the sudden swelling and discharge of a number of alveoli.^45,49,50^

Previous studies have shown that the ratio between the alveolar flow and lumen channel flow is a critical factor that determines flow patterns and recirculation.^46,51,52^ In this study, this ratio is 0.03 for the flow rate of 15 l/min in the respiratory bronchial model. Therefore, the weak flow irreversibility observed in the present simulations may be due to transient vortices. However, as these vortices last less than 1% of the entire respiratory cycle, their presence is difficult to determine experimentally. From the above discussion, it can be concluded that these flow characteristics in the alveoli contribute to the gas and aerosol mixture in the deeper lung regions.

On the other hand, these typical properties of the flow field have been also found to be the basic parameters determining the behavior of aerosol particles in the intra-acinar region.^44^ The distribution and transport of aerosol particles in the intra-acinar zone mainly take place in the higher axial velocity zones and under the influence of secondary flows. It is reported that flow instabilities affect particle deposition, especially for smaller particles.^19–21,37,53–55^

### C. Particle deposition results

**FIG. 5.**
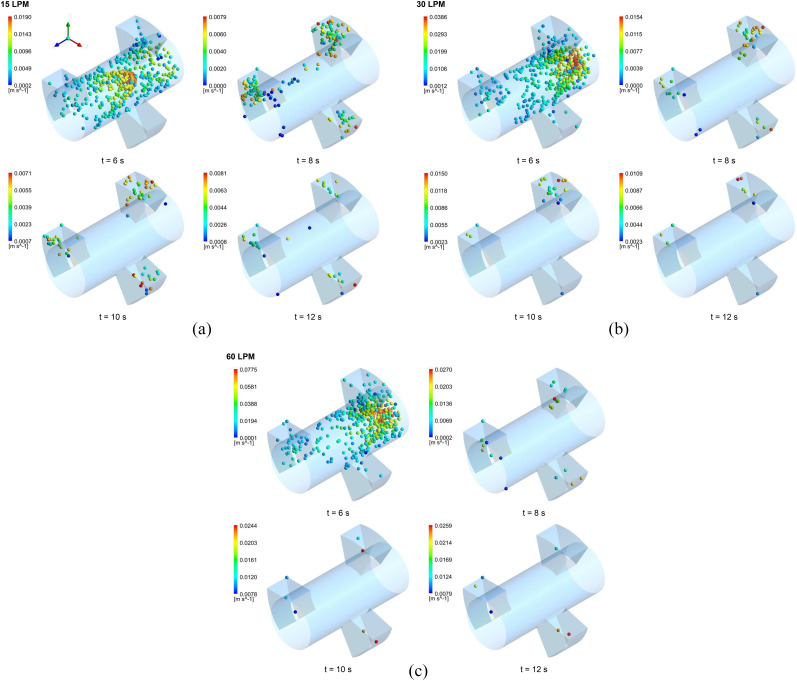
The aerosol particle positions in the respiratory bronchial model (G17) at different time intervals during the successive breathing cycles for the flow rates of (a) 15 l/min, (b) 30 l/min, and (c) 60 l/min.

The aerosol particles of 5 *μ*m in diameter were released into the flow field at the beginning of the second breathing cycle (t = 4 s), and their trajectories were simulated. Figure 5 depicts the position of the aerosol particles deposited, escaped, or remained in the suspension at different time intervals during the successive breathing cycles for the flow rate of 15 l/min with tidal volume 0.5 l. In Fig. 5(a) for G17, under the assumption that the air has a parabolic velocity profile at the model inlet, the particles also exhibit similar velocity distribution. Therefore, the particles in the center of the lumen channel move faster than those close to the channel wall (t = 6 s). While some particles moved in the axial direction along the lumen channel, others headed toward the alveolar cavities due to the cyclic alveolar wall motion induced by the respiration. At the end of the second breathing cycle (t = 8 s), the particles remaining in the suspension generally settled in the alveolar spaces and a small amount of particles remained in the lumen channel. As the multiple breathing continued, more and more particles deposited or escaped from the model, and thus, the number of particles remaining in the suspension at the end of each breath decreased gradually. Due to the subsequent cycles, the aerosol particles tend to reach the more distal regions of the lung. The particles that moved into the deeper region of the lung during the inhalation phase were attracted to the upper lung generations during the exhalation phase. The particles in the alveolar cavities were drawn into the lumen channel due to the contraction movement of the alveoli. However, as shown in Fig. 5(a), the positions of the particles during the inhalation and the exhalation phase were different due to the effect of gravity. The gravitational force during exhalation caused the particle residence times to increase, resulting in higher deposition efficiencies, especially in the distal regions. Although recent studies^18^ have shown that the direction of gravity does not affect the total deposition efficiency at the pulmonary region, the particles released in this study were slightly affected by it. Similar particle behavior was observed for the flow rates of 30 l/min and 60 l/min [Figs. 5(b) and 5(c)].

**FIG. 6.**
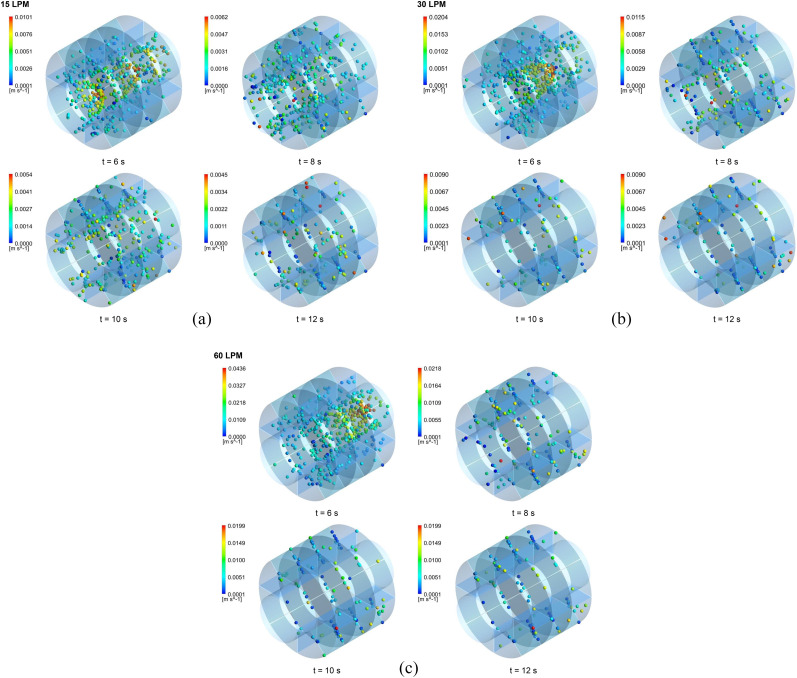
The aerosol particle positions in the alveolar duct model (G18) at different time intervals during the successive breathing cycles for the flow rates of (a) 15 l/min, (b) 30 l/min, and (c) 60 l/min.

**FIG. 7.**
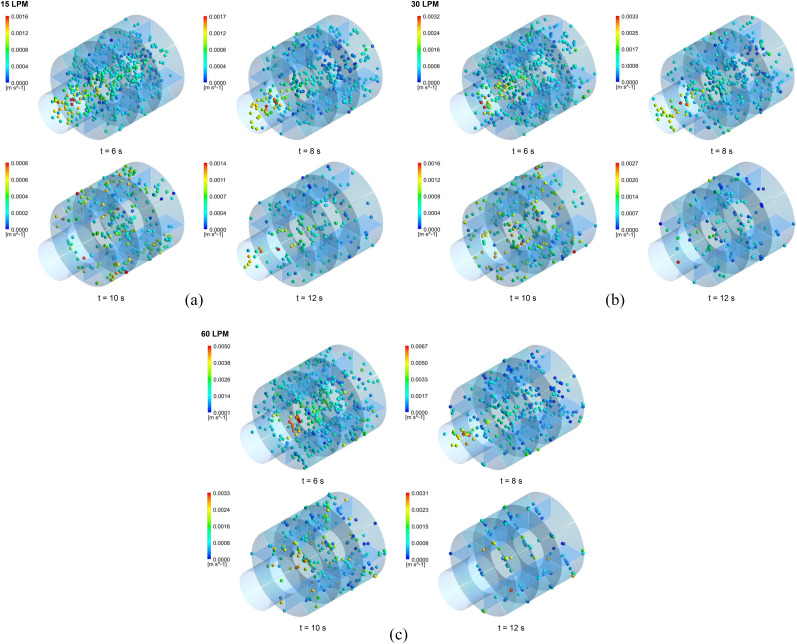
The aerosol particle positions in the alveolar sac model (G23) at different time intervals during the successive breathing cycles for the flow rates of (a) 15 l/min, (b) 30 l/min, and (c) 60 l/min.

As illustrated in Figs. 5–7, the transport and deposition patterns of aerosol particles were significantly affected by the breathing condition. As expected, the number of particles remaining in the suspension reduced with the increase in the mouth inlet flow rate. When the flow rate increased, more aerosol particles deposited or escaped from the model. Thus, more distal deposition occurred for a flow rate of 60 l/min [Figs. 6(c) and 7(c)] than for flow rates of 15 l/min and 30 l/min [Figs. 6(a), 6(b), 7(a), and 7(b)]. On the other hand, Figs. 5–7 show that more breathing cycle is required for all aerosol particles to deposit or exit in the model. Most of the particles reached full deposition or escape within two breathing cycles for the flow rate of 60 l/min, whereas some of them needed more cycles for the flow rates of 15 l/min and 30 l/min even after three breathing cycles. For the flow rate of 15 l/min, for example, the number of particles remaining in the suspension at the end of the second inhalation cycle was 0.05% and 0.016% of the total released particles for the respiratory bronchial model (G17), 0.18% and 0.053% for the alveolar duct model (G18), and 0.305% and 1.4% for the alveolar sac model (G23), respectively. This indicates that at most one breathing cycle is required for the full deposition or escape of aerosol particles.

**FIG. 8.**
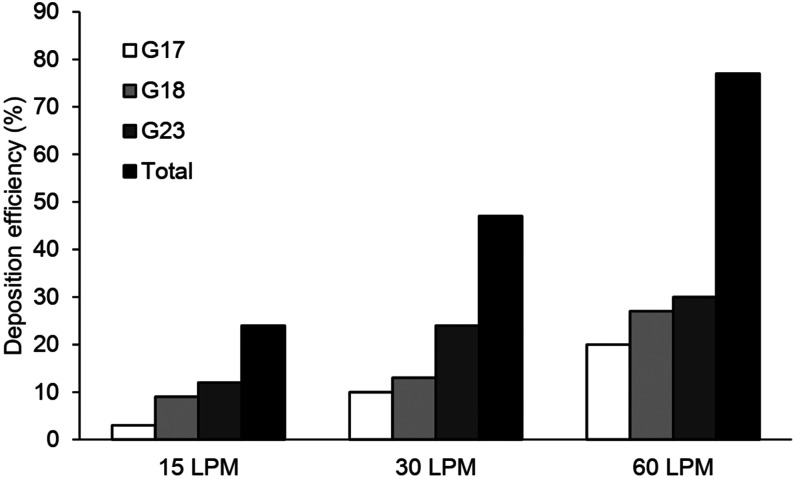
Deposition efficiency in the intra-acinar model.

The deposition efficiencies for all intra-acinar models are shown in Fig. 8. The deposition of aerosol particles for all models was strongly dependent on the respiratory conditions. The deposition efficiencies increased exponentially with the flow rate and the generation number. For the flow rate of 15 l/min, the particle deposition ranged from 3% to 12%, while the deposition varied from 10% to 24% and from 20% to 30% for the flow rate of 30 l/min and 60 l/min, respectively. In all acinar models, the total deposition efficiencies for each flow rate were 24%, 47%, and 77%, respectively. As a result, it can be said that either the type of breathing condition (i.e., light, normal, or heavy activity) or breathing cycle (i.e., single or multiple) in the intra-acinar region has a significant effect on the particle deposition.

**FIG. 9.**
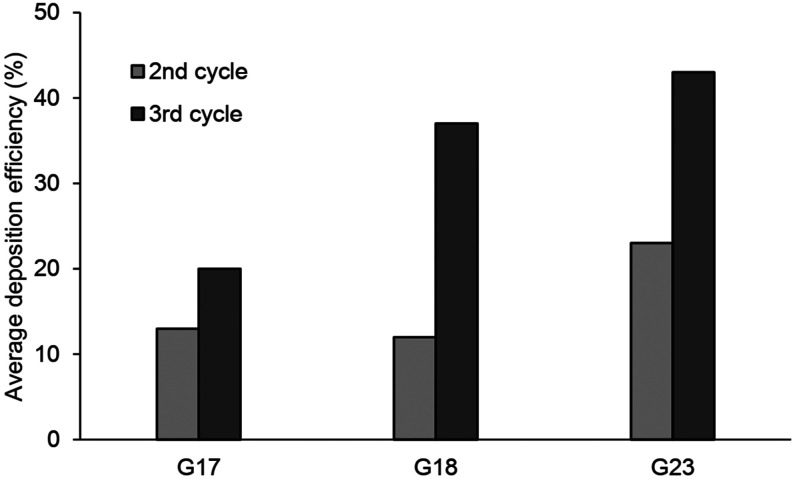
Average deposition efficiency in the second and third breathing cycles.

Heyder^56^ proposed a method to calculate the particle accumulation for infinitely long tubes. This method may apply to the state of the particles in the respiratory tract in the absence of air flow, that is, at the time of respiratory pause. However, the assumptions that they use in their models are different from the results of this study, where multiple breathing cycles and ideal sized acinar airways are taken into account. Ma and Darquenne^15^ conducted a study on the particle deposition in a 3D model of alveolated multigenerational acinar airways having spherical alveoli. Kolanjiyil and Kleinstreuer^37^ studied the airflow behavior and particle deposition in the proximal region (G16–G18), the midalveolar region (G19–G21), and the distal region (G22–G23) on a whole acinar model. It is possible to make a qualitative comparison between the studies mentioned above and the current model predictions. According to the model of Ma and Darquenne, the deposition efficiencies of the particles are 23% and 71% for the alveolar sac model and 20% and 73% for the bifurcation model. Similarly, Kolanjiyil and Kleinstreuer^37^ reported the particle deposition of 30%, 45%, and 10% for the proximal, midalveolar, and distal region, respectively. In the present study, as shown in Fig. 9, the average deposition efficiency at the end of the second and third breathing cycles (i.e., t = 8 s and t = 12 s) is about 13%–20% (33% in total) for the respiratory bronchial model, 12%–37% (49% in total) for the alveolar duct model, and 23%–43% (66% in total) for the alveolar sac model, respectively. These results can be compared qualitatively with those obtained with their particle deposition results. This again demonstrated that the multiple breathing cycles and the breathing condition played a dominant role in the deposition of particles in the pulmonary region of the lung. On the other hand, this supports the reliability of this method in which the particle deposition is determined using CFD.

At the end of the third cycle, some particles remaining in the suspension (from 0.016% to 3%) were observed; therefore, additional breathing cycles are required for an accurate estimate of the total deposition in the intra-acinar airways. It can be said that the results of the present study have a good agreement with the results of Kolanjiyil and Kleinstreuer, which reported the number of particles remaining in the suspension to be less than 0.05%. They also made a similar suggestion regarding additional breathing cycles for all aerosol particles to escape or deposit in the acinar airways. Although this cannot replace for validation, it is reasonable to assume that the current intra-acinar model provides reliable and accurate results. In the distal regions of the lung, alveoli are less and exhibit an asymmetrical structure.^57^ Because some necessary simplifications are by nature of modeling, the present and previous idealized models cannot always represent true anatomy. In addition to the common rectangular-shaped alveoli structure, spherical models,^15,58,59^ polyhedron models,^60–62^ or honeycomb-shaped alveoli^14^ have been also observed and used in modeling studies. However, it has not yet been determined how these geometrical differences between models affect the deposition of the aerosol particles in the pulmonary region.

The results in the present study also showed that the type of breathing scenarios and breathing cycle in the intra-acinar region has a significant effect on the deposition patterns of aerosol particles. In previous studies^9,10,28^ that used the rigid-walled models, it has been reported that the suspended particles can be moved deeper into the lung with the subsequent breathing cycles. In this study, the rhythmical alveolar wall motions result in the convective air flow between the alveoli and the lumen channel. Thus, most particles have deposited on the alveolar walls, while some of them have been transmitted to the deeper region of the lung. This is due to the instability in the flow direction, since the axial and the radial momentum have close values in the alveoli.^63^ However, in the proximal region (i.e., the respiratory bronchiole, G17), the suspended particles exhibited greater penetration because the axial momentum predominates in favor of the particle transport in these proximal airways. Although the number of breath periods taken into account in this study is very limited, the data obtained provide average deposition efficiencies for the intra-acinar region of the human lung. Therefore, the data obtained here can be included in the empirical models developed by Park and Wexler^11,64^ to estimate the deposition in the pulmonary region.

In this study, the deposition efficiencies are calculated as the percentage of particles that deposit on the wall of a related model by neglecting particles escaping from the model. In the case of real breathing, the particles escaping from the relevant proximal or distal airway generation may return during the breathing cycle and affect the predicted particle deposition. However, it is likely that a small part of the particles injected into the model can return. On the other hand, as shown in Fig. 9, the deposition efficiency predicted in the second respiratory cycle is not affected by the returning particles. Consequently, it should be noted that the data obtained in this study are limited to the intra-acinar region of the lung. Whether these results can be expanded for the whole tracheobronchial region of the human respiratory system has not yet been confirmed.

## V. CONCLUSIONS

In this study, idealized intra-acinar models were established to predict the aerosol particle deposition in the pulmonary region of the human lung. Three different breathing scenarios (light, normal, and heavy breathing) and the multiple breathing cycles were implemented in the CFD simulations. The results showed that the air entered into the alveoli due to the rhythmic alveolar movement and the airflow velocity here was lower than that of the lumen channel. The aerosol transport in higher generations was mainly due to the chaotic acinar flow irreversibility. The model results from the present study indicate that the transport and deposition patterns of the aerosol particles with 5 *μ*m in diameter depend on the respiration conditions in the intra-acinar region as well as the alveolar wall motion. Due to the successive breathing cycles, more particles deposit or escape to the relating acinar generation and reach the more distal regions of the lung. When the mouth flow rate is increased, the number of particles remaining in the suspension reduces, resulting in higher deposition efficiencies. The results also showed that more breathing cycle is required for full aerosol particle deposition or escape from the model. In all acinar models, the deposition fraction increased exponentially and reached to 80% with the flow rate. Considering a significant agreement among previous studies,^13,15,36,37^ the current results offer a good approach to aerosol deposition in the intra-acinar region of the human lung.

## DATA AVAILABILITY

The data that support the findings of this study are available from the corresponding author upon reasonable request.
